# Commensal Microbiota Regulate Vertebrate Innate Immunity-Insights From the Zebrafish

**DOI:** 10.3389/fimmu.2019.02100

**Published:** 2019-09-06

**Authors:** Caitlin C. Murdoch, John F. Rawls

**Affiliations:** Department of Molecular Genetics and Microbiology, Duke Microbiome Center, Duke University School of Medicine, Durham, NC, United States

**Keywords:** zebrafish, microbiota, microbiome, gnotobiotics, intestine, innate immunity, leukocyte

## Abstract

Microbial communities populate the mucosal surfaces of all animals. Metazoans have co-evolved with these microorganisms, forming symbioses that affect the molecular and cellular underpinnings of animal physiology. These microorganisms, collectively referred to as the microbiota, are found on many distinct body sites (including the skin, nasal cavity, and urogenital tract), however the most densely colonized host tissue is the intestinal tract. Although spatially confined within the intestinal lumen, the microbiota and associated products shape the development and function of the host immune system. Studies comparing gnotobiotic animals devoid of any microbes (germ free) with counterparts colonized with selected microbial communities have demonstrated that commensal microorganisms are required for the proper development and function of the immune system at homeostasis and following infectious challenge or injury. Animal model systems have been essential for defining microbiota-dependent shifts in innate immune cell function and intestinal physiology during infection and disease. In particular, the zebrafish has emerged as a powerful vertebrate model organism with unparalleled capacity for *in vivo* imaging, a full complement of genetic approaches, and facile methods to experimentally manipulate microbial communities. Here we review key insights afforded by the zebrafish into the impact of microbiota on innate immunity, including evidence that the perception of and response to the microbiota is evolutionarily conserved. We also highlight opportunities to strengthen the zebrafish model system, and to gain new insights into microbiota-innate immune interactions that would be difficult to achieve in mammalian models.

## Introduction

Evolution of multicellular life on Earth has occurred in the presence of diverse microorganisms (e.g. bacteria, fungi, viruses, and protozoa). In each animal life cycle, all exposed body surfaces are colonized by microbial communities, collectively referred to as the microbiota. Although these microbes can be found on distinct body sites, the most densely populated host compartment is the intestinal tract (1). The intestine of vertebrates is comprised of a single epithelial layer of intestinal epithelial cells (IECs) that forms a tube with a single continuous lumen allowing for the passage and absorption of dietary nutrients. The interactions in that habitat between animal hosts and their microbiota significantly shape animal biology over evolutionary and individual lifecycle time scales (2).

The detection of microbiota by host cells, including IECs, is critical for the maintenance of homeostasis and prevention of infection by pathogenic microbes (3, 4). The balance between host tolerance and inflammatory responses to commensal microorganisms is achieved through an intricate molecular dialog between microorganisms and their hosts. The innate immune system is an ancient host defense program wherein humoral and cellular components function synergistically to protect animals from infection by microorganisms. Innate immune responses occur over relatively short temporal timescales and have historically been considered to be generalizable and non-specific (5). However, there is emerging evidence challenging this dogma, whereby innate immune cells display genomic alterations that augment responses to repeated inflammatory stimuli suggestive of innate immune memory (6–8). Reactive oxygen species (ROS) in combination with secreted proteins such as anti-microbial proteins and complement factors target and kill microbes. Cellular components include myeloid cells, such as macrophages (matured from monocytes) and neutrophils, that function as professional phagocytes that engulf microorganisms and necrotic cells. While it is well-established that the microbiota promote innate immune responses in homeostasis and following injury, identification of underlying mechanisms remains a major research priority.

Microorganisms possess highly conserved molecular signatures, referred to as microbe associated molecular patterns (MAMPs). MAMPs are recognized by host cells as foreign stimuli and include the microbial surface components LPS, peptidoglycan, and flagella. Host cells detect extracellular and intracellular MAMPs through highly-conserved pattern recognition receptors (PRRs) located in both the plasma membrane and cytosol. The most thoroughly characterized PRRs include Toll like receptors (TLRs), nucleotide binding oligomerization domain (NOD) like receptors (NLRs), and RIG-I like receptors (RLRs). Host PRRs are ancient microbial sensors that have co-evolved with symbiotic commensal microbiota and pathogens. Activation of host PRRs leads to conserved signaling cascades mediated by adaptor proteins, kinases, and transcription factors, ultimately resulting in increased expression of cytokines, chemokines, and anti-microbial factors (3).

Despite being spatially compartmentalized within anatomical compartments including the intestine, oral cavity, skin, and urogenital tract, the microbiota impacts systemic host innate immune processes (9–11). Correlative studies performed in humans provide insights into the relationship between microbiota composition and specific states of disease or environmental exposure. However, mechanistic interrogation of host-microbe interactions is difficult to achieve with human studies alone, and thus animal models are essential tools for experimental manipulations. Application of gnotobiotic technologies (the ability to experimentally manipulate host-associated microbiotas) to commonly used model organisms has facilitated dissection of the influence of commensal microbes on host physiologies. To elucidate the impacts of the microbiota on host processes, animals can be derived germ-free (GF) through a variety of techniques, resulting in a sterile (or axenic) organisms devoid of microbes (12). Specific microbial strains or complex microbial consortia of interest can then be introduced to interrogate the effects of microbiota colonization on host physiology in a controlled system. Gnotobiotic mice have been instrumental in revealing the influence of microbiota on numerous aspects of host biology from development, metabolism, behavior and immunity (13). In addition to murine models, methods have been developed to rear other vertebrate and non-vertebrate animal models under gnotobiotic conditions. Fruit flies, worms, and fishes can be derived GF and subsequently colonized with a variety of microbial communities of varying composition and complexity (14). Here we review insights gained into microbiota control of vertebrate innate immunity using the zebrafish model.

### Evaluating Host-Microbe Interactions in Gnotobiotic Zebrafish

Zebrafish (*Danio rerio*) are a genetically tractable vertebrate model organism native to fresh water ecosystems of India, Burma, Bangladesh, and Nepal (15, 16). The zebrafish life cycle begins with external fertilization and embryonic development within the protective and axenic confines of the chorion, a protective membrane that is typically impermeable to microbial cells. Colonization of the developing zebrafish is thought to initially occur when larvae hatch from their chorions at approximately 3 days post-fertilization (dpf), coincident with the lumen formation of the developing intestinal tract. Zebrafish share many key anatomical and physiological features with mammalian digestive systems, including an intestinal tract comprised of differentiated absorptive and secretory epithelial cells which is capable of both absorbing diverse dietary nutrients and forming a protective barrier against lumenal factors including microbes (17, 18). 16S rRNA gene sequencing demonstrated that the zebrafish gut-associated microbiota is dominated by Proteobacteria at all developmental timepoints, although there is an expansion of Firmicutes and Fusobacteria at later adult stages (18–21). Bacterial taxa that colonize mammalian guts, such as Bacteroidetes and lactic acid bacteria (e.g., *Lactococcus lactis, Lactobacillus fermentum*, and *Weissella confusa*), have also been detected within the zebrafish intestine (18). However, reciprocal gut microbiota transplants between zebrafish and mice revealed the zebrafish intestine selects for a distinct microbial community as compared to mouse (18, 22). Like mammalian intestinal microbiotas, the composition of zebrafish intestinal microbial communities is responsive to dietary perturbations and varies substantially across aquaculture facilities (21, 23–25).

Methods to derive zebrafish embryos germ free (GF) and their subsequent rearing under gnotobiotic conditions are well-established (26, 27). While gnotobiotic husbandry of murine model organisms is technically challenging and expensive, zebrafish can be maintained germ-free or colonized with defined bacterial strains or communities (conventionalized—CV) with ease and at relatively low cost (Table 1). Briefly, zebrafish embryos residing within the axenic environment of a protective chorion are passaged through a series of antibiotic, iodine, and bleach baths that sterilize the surface of the chorion. Subsequently, those derived zebrafish are housed in sterile media and provided sterilized diets when warranted. Microbial consortia, strains, or their products can be added to the housing media to test their impact on host biology or their ecology in association with the host. Inoculation of GF zebrafish larvae with complex bacterial communities or defined strains demonstrated that even some mammalian commensal taxa, such as *Escherichia, Enterococcus, Bacillus, Roseburia*, and *Prevotella*, can colonize zebrafish larvae (22, 42), thus enabling the use of “humanized” zebrafish to for medium-throughput investigation of host-microbiota interactions. Furthermore, probiotic bacterial strains including several species of *Lactobacilli*, colonize zebrafish and can influence host immune responses and outcomes to bacterial infection (37–39, 43, 44). Over the last 15 years, these foundational insights and methods have allowed the zebrafish to become established as a valuable new vertebrate model for investigating host-microbiota interactions.

**Table 1:** Microbial specific effects on zebrafish innate immunity.

**Microbial taxa or product**	**Phenotype**	**Tissue**	**References**
Complex microbiota (CV)	Increased neutrophil recruitment vs. GF	Gut	(28, 29)
	Increased systemic abundance of neutrophils	Whole 6 dpf larvae	(28, 29)
	Increased neutrophil velocity	Gut, CHT, fin	(28)
	Increased neutrophil recruitment to tail wound;	Caudal fin	(28, 30, 31)
	Increased NF-κB signaling	Gut, swim bladder	(32)
	Increased pro-inflammatory mRNAs	Dissected digestive tissue	(22, 32)
	Increased *intestinal alkaline phosphatase* expression and LPS detoxification	Dissected digestive tissue	(33)
*Shewanella* sp. ZOR0012	Gut CFU negatively correlated to neutrophil number	Gut	(29)
*Shewanella* sp. ZWU0012 (previous name T1E1C05)	Gut CFU negatively correlated to neutrophil number	Dissected digestive tissue	(22)
*Aeromonas* sp. ZWU0008 (previous name T1E1A06)	Increased pro-inflammatory mRNAs	Dissected digestive tissue	(22)
*Aeromonas* sp. ZOR0001	Increased neutrophil recruitment vs. GF	Gut	(29)
*Vibrio* sp. ZWU0020	Increased neutrophil recruitment vs. GF	Gut	(29)
*Acinetobacter* sp. ZOR0008	Decreased neutrophil number vs. CV	Gut	(29)
*Plesiomonas* sp. ZWU0015 (previous name T1N1D03)	Increased pro-inflammatory mRNAs	Dissected digestive tissue	(22)
*Plesiomonas* sp. ZOR0011	No difference in neutrophil recruitment vs. GF	Gut	(29)
*Enterobacter* sp. ZOR0014	No difference in neutrophil recruitment vs. GF	Gut	(29)
*Delftia* sp. ZNC0008	Decreased neutrophil number vs. CV	Gut	(29)
*Variovorax* sp. ZNC0006	Decreased neutrophil number vs. CV	Gut	(29)
AimA (secreted from *Aeromonas* sp.)	Dampens neutrophil recruitment	Gut	(34)
*Exiguobacterium acetylicum* ZWU0009	Increased expression of genes involved in (1) cell matrix adhesion and (2) response to bacterium	Whole animal	(35)
*Chryseobacterium sp*. ZOR0023	Increased expression of genes involved in (1) negative regulation of cell proliferation, (2) cell-cell adhesion, (3) regulation of MAPK activity, (4) apoptotic process, (5) activation of endopeptidase, (6) transcription	Whole animal	(35)
*Aeromonas hydrophila* ATCC35654	Increased expression of pro-inflammatory mRNAs	Dissected digestive tissue	(18, 22)
*Pseudomonas aeruginosa* PAO1	Increased expression of pro-inflammatory mRNAs (*saa* and *mpx*)	Dissected digestive tissue	(22)
*P. aeruginosa* PAK	Increased NF-κB activation	Gut, liver, swim bladder, muscle, whole animal	(32, 36)
*P. aeruginosa* ΔfliC PAK	No significant NF-κb activation; increased NF-κb activation	Gut, whole animal; swim bladder, muscle	(32, 36)
*P. aeruginosa* ΔmotACBD PAK	No significant NF-κb activation; increased NF-κb activation	Gut, whole animal; liver and muscle	(32, 36)
*P. aeruginosa* LPS	Increased expression of pro-inflammatory mRNAs (*saa* and *mpx*)	Dissected digestive tissue	(22)
*Escherichia coli* MG1655	Increased expression of pro-inflammatory mRNAs (*saa* and *mpx*)	Dissected digestive tissue	(22)
*Staphylococcus* sp. MWU0002 (previous name M2EA04)	No significant increase in pro-inflammatory mRNAs vs. GF (*saa* and *mpx*)	Dissected digestive tissue	(22)
*Enterococcus* sp. MWU0002 (previous name M2E1F06)	No significant increase in pro-inflammatory mRNAs vs. GF (*saa* and *mpx*)	Dissected digestive tissue	(22)
*Citrobacter* sp. ZWU0013 (previous name T1E1C07)	Increased expression of pro-inflammatory mRNAs (*saa* and *mpx*)	Dissected digestive tissue	(22)
*Lactobacillus casei* BL23	Protective against *Aeromonas veronii* infection, increased expression of *tnfa, il1b, il10*, and *saa*	Whole animal	(37)
*Lactobacillus plantarum* ST-III	Protects against toxic effect of triclosan by mediating microbiota composition	Gut	(38)
*Lactobacillus rhamnosus* IMC 501	Increased expression of pro-inflammatory mRNAs *il1b* and *tnfa*; modulates commensal microbiota and host gene expression of lipid metabolism genes	Dissected adult intestine; larval gut	(39, 40)
*Lactobacillus casei* BL23 EPS	Protects against high fat diet induced hepatic steatosis	Liver	(41)
*Lactobacillus rhamnosus* GG EPS	Protects against high fat diet induced hepatic steatosis	Liver	(41)

### Zebrafish as a Model to Study Innate Immunity

In parallel to its advancement as a model for host-microbiota interactions, the zebrafish has also emerged as a powerful vertebrate model for the study of innate immunology. The zebrafish offers several advantages for such studies, including a fully sequenced genome and a complement of genetic tools which facilitate transgenesis and mutagenesis. Zebrafish possess hematopoietic lineages that are highly-conserved with those of mammals which function analogously to counteract inflammatory stimuli and maintain host heath (45). The zebrafish genome encodes a number of TLRs and NLRs that are expressed in diverse cell and tissue types where they respond to inflammatory stimuli (46). Surveillance of the extracellular and intracellular environments by these host PRRs functions to activate immune pathways and promoting immunological tolerance or inflammatory responses following microbial stimulation or tissue injury.

Prior to 4 weeks of age, zebrafish do not possess a fully functional adaptive immune system (47). The innate immune system plays a pivotal role during this period, functioning as the sole defense against microbial invasion in zebrafish larvae. This affords a unique opportunity to study the innate immune system in isolation, which cannot be easily achieved in mammalian models. Early in development (~22–33 h post fertilization), primitive immune cells are derived from the yolk sac and the intermediate cell mass ICM (analogous to the mammalian yolk sac) (48). Definitive hematopoiesis begins approximately at 1 dpf, giving rise to multiple lineages of immune cells including neutrophils and macrophages. HSCs (hematopoietic stem cells; *runx-, c-myb-*, and/or *cd41-*positive) travel from the aorta-gonad-mesonephros (AGM) to populate the caudal hematopoietic tissue (CHT), kidney, and thymus (49). Myeloid cells including monocytes, macrophages, neutrophils, mast cells, dendritic cells and eosinophils have been described in zebrafish (50–54) (Table 2). Cytological, transcriptomic, and functional analyses of zebrafish myeloid cells indicate that these populations function analogously to mammalian counterparts (45). Importantly, the optical transparency of zebrafish larvae offers un-paralleled opportunities to use transgenic reporters for assessing spatiotemporal leukocyte responses to a variety of inflammatory stimuli in ways that are not feasible in mammalian models.

**Table 2:** Myeloid lineages described in zebrafish.

**Innate immune cell type**	**Promoter/BAC transgenic element**	**Antibody**	**Histochemical stain**	**References**
Neutrophil	*lyz, mpx*	Mpx; L-plastin	Sudan black; myeloperoxidase; periodic acid–Schiff, toluidine blue, Wright-Giemsa (WG)	(55, 56)
Macrophage	*mpeg1, mfap4, irg1*	L-plastin	Neutral red; Wright-Giemsa (WG)	(57, 58)
Dendritic cell	*mpx*	N/A	Wright-Giemsa (WG); peanut agglutinin (PGA)	(53)
Mast cell	*cpa5*	Anti-human FcεRIγ	Hematoxylin and eosin, periodic acid–Schiff, toluidine blue, KIT, tryptase, Wright-Giemsa (WG)	(52, 59, 60)
Eosinophil	*gata2* (EGFP-hi)	N/A	Periodic acid-Schiff, myeloperoxidase, toluidine blue, Wright Giemsa	(54)
HSC	*cd41; runx1; gata2b*	N/A	N/A	(61)

As described in the following sections, studies in gnotobiotic zebrafish have revealed highly-conserved innate immune responses to microbiota colonization. Although not detailed in this review, the commensal microbiota further elicits alterations in zebrafish development, behavior, and metabolism analogous to mammalian counterparts, underscoring the utility of the zebrafish as a tractable model to study host-microbe interactions (18, 19, 22, 33, 62–64). Additionally, although not discussed here, zebrafish models of infectious disease have also provided critical insights into the mechanisms of host-pathogen interactions. In particular, numerous studies using the natural fish pathogen *Mycobacterium marinum* (which is closely related to the *Mycobacterium tuberculosis* complex), have identified both host and bacterial mechanisms that underlie disease severity which are conserved with human tuberculosis (65, 66).

### Microbiota Influences Steady State Innate Immunity in Zebrafish

**Figure 1:**
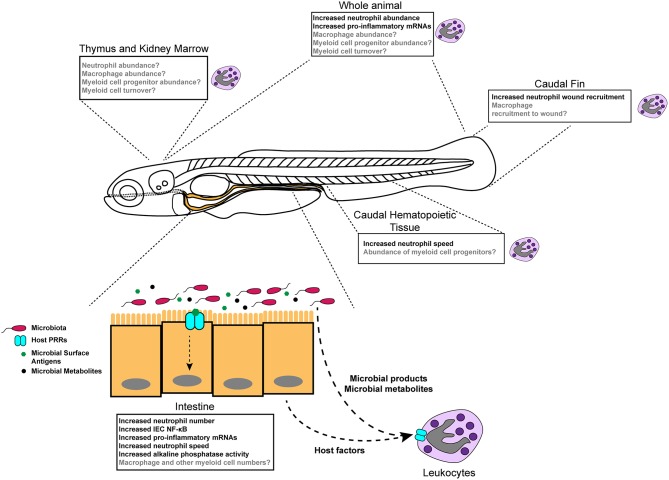
Diverse effects of the microbiota on innate immune development and function in zebrafish. Colonization of germ-free zebrafish larvae with microbiota stimulates inflammatory gene expression (detected in specific tissues and whole larvae), neutrophil behavior and activity, neutrophil abundance in homeostasis, and neutrophil mobilization to injury. Boxes indicate different tissue-specific phenotypes that are known to be (black text) or are possibly (gray text) affected by microbiota.

Colonization of zebrafish larvae with a complex microbiota stimulates innate immune responses that have also been observed in mammals, underscoring the importance of these responses over evolutionary timescales (18, 19, 22) (Figure 1, Table 3). Innate immune activation by commensal microorganisms is achieved, in part, through host gene expression programs. Studies in mice have reported microbiota-dependent host transcriptional responses in intestinal epithelia, including marked induction of genes encoding cytokines, chemokines, anti-microbial peptides, and other inflammatory mediators (17, 62, 67, 69, 95, 96). Similarly, facile gnotobiotic manipulations in zebrafish larvae have allowed for investigation into the impact of specific bacterial strains on host innate immune signaling more quickly and with greater scalability than comparable experiments in murine models. Application of mono- and poly-association studies in gnotobiotic zebrafish larvae have illustrated microbe-specific impacts on host innate immunity (Table 1) (22, 29, 35). Our transcriptomic analyses of digestive tracts of germ-free and conventionalized zebrafish larvae revealed many genes are regulated by microbiota. Moreover, many microbiota-responsive zebrafish genes have mouse homologs that are also similarly transcriptionally responsive to microbiota colonization in the intestine, indicating that host responses to the microbiota are deeply conserved during vertebrate evolution (18). Notably, innate immune genes involved in anti-microbial peroxide production and signaling to leukocytes, *myeloperoxidase* (*mpx*) and *serum amyloid a* (*saa*), exhibited dynamic and variable transcriptional induction in zebrafish digestive tracts following colonization with complex microbiota and individual bacterial taxa. Some bacteria including *Pseudomonas aeruginosa, Aeromonas hydrophila*, and *Escherichia coli* strains potently induced *mpx* and *saa* transcripts, whereas other strains including *Shewanella* sp. and *Staphylococcus* sp. failed to induce transcription of these same genes. Further, complex communities of microbes isolated from zebrafish digestive tracts more potently stimulated expression of these innate immune markers compared to monoassociation with individual strains (22). Finally, the presence or absence of sterilized diet significantly affected several of these host responses, underscoring an intimate and complex relationship between host, microbiota, and nutrient availability (22).

**Table 3:** Conservation of microbiota-induced innate immune phenotypes in zebrafish and mice.

**Cell/tissue type**	**Phenotype**	**Zebrafish**	**Mice**	**References**
Intestine	Increased pro-inflammatory mRNAs	X	X	(17, 67–69)
	Increased alkaline phosphatase activity	X		(19, 33)
	Increased nutrient absorption	X	X	(17, 67–72)
	Exacerbation of intestinal injury	X	X	(20, 73–78)
	Increased immune cell infiltration	X	X	(19, 28, 79)
	Increased proliferation	X	X	(19, 64, 80)
Myeloid Cells	Increased pro-inflammatory mRNAs	X	X	(28, 31, 81–83)
	Increased bacterial killing activity		X	(11, 84–87)
	Increased longevity		X	(88, 89)
	Increased systemic abundance	X	X	(28, 90)
	Increased abundance in hematopoetic compartments		X	(6, 89–93)
	Increased recruitment to wounds	X	X	(28, 31, 94)
	Increased velocity and directional migration	X		(28)
Whole animal	Protection against systemic microbial infection	X	X	(30, 90, 92)

Motivated by previous monoassociation studies that identified individual bacterial taxa that differentially promote zebrafish lipid metabolism (72), a recent report assessed whole animal transcriptional responses to bacterial colonization of representative Firmicutes (*Exiguobacterium acetylicum*) and Bacteriodetes (*Chryseobacterium* sp.) commensal species. This study identified 65 genes that were uniformly differentially expressed in zebrafish either monoassociated with the commensal strains *E. acetylicum* or *Chryseobacterium* sp., or colonized by complex microbiota. These findings in whole zebrafish larvae, taken together with previous analyses of digestive tracts from monoassociated larvae, suggest that there may be shared host transcriptional responses evoked by colonization with diverse microbial taxa, although this remains to be determined with a more extensive panel of bacteria (35). Collectively, these findings demonstrate that specific bacterial strains in zebrafish are sufficient to elicit innate immune responses, and that complex microbial communities synergistically affect host innate immunity (Table 1).

A major challenge in understanding transcriptional regulatory networks that mediate host responses to microbiota is identification of the specific underlying transcription factors. Many innate immune genes are regulated by the highly conserved NF-κB transcription factor complex (97). Due to the optical transparency of zebrafish larvae, high resolution *in vivo* imaging of transcriptional reporter transgenes for key transcription factors provide unique opportunities to understand the spatiotemporal dynamics of microbiota-responsive signaling circuits. Our analysis of gnotobiotic zebrafish larvae harboring a transgenic NF-κB reporter, *Tg(NF*κ*B:EGFP)* ^*nc*1^, demonstrated that microbial signals induce NF-κB signaling in a variety of cell and tissue types in larval zebrafish, including absorptive and secretory cells within the intestine (32). Further analysis of intestinal NF-κB reporter dynamics demonstrated that this pathway is activated in part by bacterial flagellar motility. *P. aeruginosa* mutant strains lacking flagellar components or flagellar function failed to induce the NF-κB transgene in the intestine or increase expression of innate immune genes, *saa* and *mpx* in dissected digestive tracts (32, 36). Moreover, pharmacological and genetic inhibition of NF-κB/MyD88 signaling in zebrafish larvae abrogated the microbiota-dependent induction of innate immune genes including *complement factor b* (*cfb*) and *saa* (32). Microbiota-dependent stimulation of NF-κB in the intestine of zebrafish likely shapes host innate immunity. Genetic tools, including *myd88* mutants and PRR mutants, will provide key insights of the microbial signaling pathways that lead to innate immune responses mediated by commensal microbiota in zebrafish (35, 98).

MyD88 is an evolutionarily conserved TLR adaptor protein that initiates inflammatory signaling pathways following ligand recognition, ultimately leading to transcriptional induction of innate immune genes. Zebrafish genetically deficient in *myd88* are susceptible to acute infection by bacterial pathogens *Edwardsiella tarda* and *Salmonella typhimurium* (98). Findings from this work also demonstrated that expression levels of innate immune genes, including *il1b* and *mmp9*, as well as transcription factors, AP-1 and NF-κB, depend upon *myd88*. Moreover, gene expression analysis of whole larvae revealed that *myd88*, at least in part, mediates host sensing of MAMPs, Flagellin and LPS in zebrafish. Interestingly, *myd88* deficiency had no impact on the abundance of leukocyte populations in zebrafish larvae (mpx:GFP^+^ neutrophils and l-plastin^+^mpx:GFP^−^ macrophages) (98), which might suggest that the microbiota mediate leukocyte phenotypes via MyD88-independent mechanisms. However, this analysis was performed in 3 dpf larvae, coincident with the timepoint of canonical microbiota colonization. Indeed, a more recent report documented a decrease in the abundance of myeloperoxidase-positive intestine-associated neutrophils in *myd88* mutant larvae at 6 dpf (99). This underscores that the impact of microbiota on host innate immunity and the specific bacterial and host mechanisms mediating those interactions, may vary as a function of host developmental stage.

### Intestinal Epithelial Cells (IECs) Regulate Systemic Innate Immunity in Response to Microbiota Colonization

Since the largest collection of microbes in the animal body typically reside in the intestine, the intestinal epithelium represents a large and critical component of the innate immune system. The intestine is constantly stimulated with environmental factors from both diet and the complex microbial community residing within the lumen. To maintain a symbiotic relationship with the gut microbiota, IECs must detect bacterial signatures and respond appropriately to modify host physiology. IECs are a critical node in the host circuitry which transduces information regarding the state of the microbiota to other distal tissues and cell types, including innate immune cells (4, 95). As such, IECs are important modulators of host innate immunity and dynamically respond to microbiota colonization in a number of ways detailed below.

One mechanism by which IECs coordinate responses to environment stimuli is through transcriptional programs. Gene expression is regulated through the action of transcription factors (TFs) that bind to *cis-*regulatory elements (CREs) in enhancer and promoter regions throughout the genome. Active enhancer regions are often situated in regions of accessible chromatin which, depleted of nucleosomes, allows for TF binding and transcriptional regulation. Active or poised enhancers can be identified in regions of accessible chromatin marked by specific nucleosome post-translational modifications including acetylation and methylation at conserved lysine residues (e.g., H3K27ac or H3K4me1) (100). Upon TF binding in an enhancer, transcription is either activated or suppressed, resulting in alterations in gene expression. Our genome-wide comparisons of gene expression and accessible chromatin in IECs from zebrafish, stickleback, mouse, and human recently revealed a conserved transcriptional network that has been conserved since their last common ancestor 420 million years ago (17). Notably, several TFs included in this conserved transcriptional network have known associations with the human Inflammatory Bowel Diseases (IBD) (17), which are known to be driven in part by aberrant interactions with the microbiota. Analysis of microbiota-responsive transcriptional regulatory pathways in zebrafish therefore have the potential to inform our understanding of host-microbiota interactions in humans and other mammals.

Microbiota colonization of the vertebrate intestine induces robust changes in IEC gene expression programs as observed in mice and zebrafish (17, 67–69, 101), yet does not significantly alter overall chromatin accessibility in mice (62). These data suggest that microbiota-dependent changes in intestinal gene expression does not rely on alterations to chromatin accessibility, and is instead likely driven by differential activity of TFs and enhancers. The identification of genomic mechanisms that mediate IEC responses to colonization is an active area of investigation. For example, our recent work in zebrafish and mice has identified Hepatocyte Nuclear Factor alpha (HNF4A) as an evolutionarily conserved TF that coordinates global changes in gene expression following microbial colonization (67). Mutation of *Hnf4a* in mouse IECs leads to intestinal inflammation (102) and genetic variants at human *HNF4A* have been linked to inflammatory bowel diseases (102–106). Analysis of enhancer activation in small intestinal IECs from gnotobiotic mice using chromatin-immunoprecipitation (ChIP) sequencing for acetylation marks associated with active enhancers (H3K27ac) revealed that microbiota colonization was correlated with activation of enhancers linked to microbially-induced genes, and deactivation of enhancers linked to microbially-suppressed genes (67). Interestingly, these microbiota-responsive enhancers in mice were enriched for binding motifs for several of the TFs previously identified in the conserved IEC transcriptional network shared between fishes and mammals such as HNF4, STAT, IRF, and ETS factor TFs (17). Collectively, these data indicate that the microbiota influence multiple conserved gene-regulatory mechanisms which ultimately affect host gene-expression programs and may contribute to inflammatory diseases.

Recognition of microbially-derived signals and metabolites through PRRs expressed in IECs contributes to the host immune response. Gene Ontology (GO) analysis of up-regulated genes in IECs (and dissected digestive tracts) following microbiota colonization include an over-representation of innate immune and defense response pathways in mice and zebrafish (17, 62, 67). More specifically, the intestinal microbiota promote expression of complement factors, anti-microbial peptides (AMPs), chemokines, and cytokines (22, 28, 32, 62, 67, 68). The coordinated transcriptional up-regulation of these secreted immunomodulatory factors following microbiota colonization shapes local and systemic innate immunity.

Several cytokines, chemokines, and other immunomodulatory proteins are constitutively expressed or induced in IECs following microbiota colonization (62, 67, 68, 107–112). As these host factors are secreted, they can have both autocrine and paracrine effects on adjacent cells or tissues. As many innate and adaptive immune cell types reside within the mammalian intestinal lamina propria (LP) as well as within/around the zebrafish intestine, IEC-derived cytokine factors shape the activation and function of the local immune cell milieu and promote proper immune development during homeostasis (95). In mice, IEC transcription of the inflammasome-dependent cytokines *Il1B* and *Il18* is induced following microbiota colonization (67). These cytokines promote innate immune cell recruitment and epithelial homeostasis (113–115). In zebrafish, microbiota-induced expression of the secreted apolipoprotein Saa in IECs promoted neutrophil recruitment to the gut while mediating systemic immune responses to bacterial infection and injury (31). Thus, IECs exhibit dynamic transcriptional responses to microbiota-derived signals in order to regulate local and systemic immunity.

### Microbiota Influences Neutrophil Activity in Zebrafish

Commensal microbiota regulate innate immune cell development and function, which has important implications for health and disease. Neutrophils are the most abundant white blood cell in circulation in humans, and are typically the first innate immune cell type recruited to sites of injury or infection. Neutrophils are professional phagocytes that promote the clearance of microorganisms and cellular debris through a variety of mechanisms (116–118). Numerous reports from murine models highlight that neutrophil functions are mediated by microbiota colonization (11). In zebrafish, neutrophils have been identified and specific transgenic markers exist, including *Tg(lyz:EGFP)*^*nz*117^ and *TgBAC(mpx:EGFP)*^*i*114^ (56, 119) (Table 2). Similar to studies in mammalian models, microbiota colonization of larval zebrafish alters neutrophil behavior, intestinal infiltration, and transcriptional activation in homeostasis (19, 28, 30, 31, 33, 35). Our time-lapse imaging of neutrophils in GF and CV larvae demonstrated that neutrophils move with higher speed in tissues including the CHT, intestine, and the fin of colonized hosts. This neutrophil behavior is in part mediated through microbiota-induced secreted immunomodulatory protein Saa (28, 31). Moreover, qPCR analysis of pro-inflammatory mRNAs from sorted neutrophils illustrated that transcripts encoding cytokines (*tnfa*), ROS-producing enzymes (*mpx* and *ncf1*), and anti-microbial peptides (*pglyrp2* and *pglyrp5*) were more highly expressed in the presence of a microbiota (28, 31). These data suggest that the commensal microbiota elevate the inflammatory tone of neutrophils in zebrafish at homeostasis, which is associated with behavioral differences.

In addition to affecting multiple aspects of neutrophil biology systemically and in distal tissues, the microbiota also promote intestinal infiltration of neutrophils in zebrafish larvae. Colonized zebrafish larvae display augmented numbers of intestine-associated neutrophils relative to GF controls (28, 30, 33, 35). Studies using morpholino knockdown approaches revealed that *myd88* and *tnfr1* signaling promoted neutrophil recruitment to the gut in colonized zebrafish larvae (33). These data suggest that microbiota-derived MAMPS in the intestine signal through PRRs via MyD88 to potentiate neutrophil recruitment to the gut.

To assess the impact of specific bacterial species on intestinal neutrophil recruitment, gnotobiotic zebrafish larvae were monoassociated with a panel of zebrafish gut bacterial isolates and intestinal neutrophil numbers quantified (29). This work demonstrated that specific bacterial isolates, such as *Aeromonas* sp. and *Vibrio* sp., promoted higher levels of neutrophil abundance in the gut compared to strains like *Shewanella* sp. Furthermore, di-associations with pairwise combinations of these same strains revealed that the dominant species (i.e., the most abundant as determined by CFU plating) did not always positively correlate with intestinal neutrophil recruitment. Findings from this study revealed that *Shewanella* sp. secreted a factor that suppressed gut neutrophil recruitment. A subsequent study determined that *Aeromonas* sp. secreted an immunomodulatory protein “AimA” which prevented aberrant intestinal neutrophil recruitment (34). Together, these data demonstrate that specific microbial species can influence host innate immune cells independent of bacterial abundance within the gut, effects which may be mediated in part by secreted immunomodulatory factors or presentation of distinct surface antigens (Table 1).

Neutrophil abundance is elevated in colonized zebrafish larvae relative to GF, yet, it remains unclear if this is due to increased rates of production or longevity of neutrophil populations (28). In mice, the microbiota influence hematopoietic lineages at multiple stages of differentiation and commitment (9). It has been shown in murine models that bacterial products, such as peptidoglycan, signal to distal tissues including progenitor cells in the mammalian primary hematopoietic compartment, the bone marrow (BM) (91). Interestingly, the complexity of host associated microbiotas is positively correlated with the enhancement of steady state myelopoiesis (91). Studies in mice have further demonstrated that microbiota colonization is associated a with TLR4-MyD88 dependent increase in the abundance of not only BM HSCs, but also BM granulocyte and monocyte progenitors (GMPs), which positively correlates with increased numbers of BM and circulating neutrophils and monocytes (90, 92, 93). Exposure of GF mice to MAMPs is sufficient to promote differentiation of GMPs to myeloid cells, suggesting that microbial products or downstream signals expand the BM myeloid cell pool (90). Moreover, the microbiota promote circulating neutrophil longevity via a TLR2/4-MyD88 signaling axis (89). Collectively, these findings from murine models indicate that the abundance of BM and circulating myeloid cells are elevated through host detection of microbiota-derived molecular cues by PRRs. It is likely that the commensal microbiota influence myeloid cells at several stages of differentiation and development in zebrafish, but, in the absence of transgenic markers for GMPs or other precursors, this remains to be determined.

### Microbiota Influence Innate Immune Responses to Peripheral Injury and Inflammation in Zebrafish

Following injury or infection, the concerted actions of innate immune cells (including macrophages, neutrophils, and dendritic cells) play critical roles in clearing pathogens, removing necrotic tissue, signaling to other cell-types, enhancing wound healing, and ultimately restoring homeostasis through a variety of mechanisms. Immediately following injury, local production of cytokines and inflammatory mediators initiates signaling cascades that recruit both circulating and tissue-resident immune cells to sites of injury (118). The ability to image leukocyte populations (such as neutrophils and macrophages) and interrogate their functions *in vivo* provides a novel platform to rigorously investigate the mechanisms underlying innate immune cell functions. In zebrafish, amputation of the caudal fin leads to a gradient of ROS, damage associated molecular patterns (DAMPs), and MAMPs which leads to leukocyte recruitment to the tail wound margin (30, 120–123). Studies in many vertebrate animal models have demonstrated that host-associated microbiota promotes the recruitment of innate immune cells to sites of trauma. Colonization by complex intestinal microbiota augmented neutrophil recruitment to a caudal fin amputation (28, 30, 31). Moreover, expression of the pro-inflammatory mRNAs *il1b* and *ccl-c25ab* were significantly elevated in wounded larvae colonized with a microbiota as compared to GF wounded larvae (30). This suggests that microbial signals are necessary for proper neutrophil mobilization in response to peripheral injury. Similarly, experiments in murine models demonstrated that neutrophil extravasation and recruitment following intraperitoneal injection of the chemoattractant zymosan is reduced in both GF mice and antibiotics treated mice as compared to specific pathogen free (SPF) in a *Myd88*-dependent manner (94). To our knowledge, the influence of the microbiota on macrophage recruitment to injury has yet to be described.

Perturbation of the intestinal microbiota is associated with intestinal inflammation and is thought to underlie the pathology of chronic intestinal diseases such as IBD. Indeed, many genetic mouse models of IBD, such as *Il10*^−/−^, require microbiota colonization to develop spontaneous colitis (74). Aspects of human IBD can also be modeled in zebrafish. For example, *uhrf1* mutant zebrafish larvae develop intestinal inflammation characterized by increased intestinal permeability, reduced epithelial thickness, and increased intestinal levels of the pro-inflammatory mRNA *tnfa* (75). Interestingly, GF *uhrf1* mutant larvae exhibit attenuated intestinal *tnfa* expression, demonstrating that the commensal microbiota exacerbate intestinal inflammation in this model. Similarly, pro-inflammatory effects of the microbiota have been documented in zebrafish using chemical models of intestinal injury including oxazolone (20), Trinitrobenzenesulfonic acid (TNBS) (77), dextran sodium sulfate (DSS) (76), soy saponin (124), and Glafenine (73) exposures. Zebrafish exposed to these chemical agents exhibited various manifestations of intestinal inflammation, including increased expression of pro-inflammatory mRNAs and myeloid cell infiltration to the gut, which were attenuated in animals reared GF or treated with antibiotics (20, 73, 76, 77, 124). Collectively, these studies demonstrate an evolutionarily conserved pro-inflammatory effect of the microbiota following genetic and chemical insult to the intestine.

The microbiota promote the development of the innate immune system, which facilitates enhanced pathogen elimination following infection. In zebrafish, GF larvae were more sensitive to viral infection by spring viremia of carp virus (SVCV), having higher mortality rates and decreased expression of anti-viral transcripts compared to conventionally raised zebrafish larvae (30). In mammals, the microbiota protects the host against systemic infection from bacterial pathogens (including *Escherichia coli, Klebsiella pneumoniae*, and *Listeria monocytogenes*) by accelerating production of circulating and BM neutrophils and monocytes (90, 92). In antibiotics-treated mice, reduced plasma concentrations of the granulopoietic cytokine G-CSF may explain the failed granulocytic response following bacterial infection (92). Products derived from the microbiota can also mediate the killing activity of host BM myeloid cells. Microbial products, such as peptidoglycan, have been detected in the serum of colonized mice, suggesting that microbial factors can traverse the intestinal epithelium and enter circulation (86). Detection of microbiota-derived peptidoglycan by NOD1 enhanced BM neutrophil phagocytosis of *Streptococcus pneumoniae* and *Staphylococcus aureus* (86). Technical hurdles in zebrafish, including their relatively small size and the current lack of transgenic reporters labeling myeloid progenitor cell populations, has thus far prevented analysis of microbiota-dependent impacts on the differentiation, abundance, and activities of myeloid cell populations within hematopoietic compartments.

### Host Strategies to Control Resident Microbiota and Innate Immune Tolerance in Zebrafish

Collectively, the intestinal microbiota promotes increased numbers of myeloid cells (in circulation and the BM of mice; in the intestine and whole animal in zebrafish) as well as their bactericidal activity (11). However, the host must balance this response to temper excessive innate immune system activation. While we observe elevated pro-inflammatory signaling following microbiota colonization, there is also a concomitant activation of anti-inflammatory pathways. It has recently become appreciated that there are host mechanisms to limit aberrant activation of the innate immune system by the microbiota. As detailed below, work in zebrafish has provided novel insights into mechanisms of immune tolerance to the microbiota. By integrating powerful genetic manipulations with facile gnotobiotics in zebrafish, we recently demonstrated that the host apolipoprotein Serum amyloid a (Saa) was induced following microbiota colonization and suppressed neutrophil bactericidal activity and expression of pro-inflammatory mRNAs, thus restricting aberrant activation (31).

Colonization of the intestine by commensal bacteria is accompanied by a concomitant increase in the lumenal concentration of LPS within the intestine. To counteract the pro-inflammatory and potentially toxic effects of increased LPS signaling, intestinal alkaline phosphatase activity increases to detoxify LPS (19). In zebrafish larvae, expression of *intestinal alkaline phosphatase* (*iap*) is induced in IECs following bacterial colonization in a Myd88 dependent manner, and restricted excessive neutrophil recruitment to the gut following microbial colonization (33). IAP was subsequently shown to also play important roles in gut mucosal homeostasis in mice (125), illustrating the ability of the zebrafish to uncover novel and conserved mechanisms underlying host-microbiota interaction.

Further work has shown that the enteric nervous system (ENS) promotes intestinal homeostasis by controlling the composition of the microbiota. Zebrafish larvae with a mutation in *sox10*, a gene associated with Hischsprung's disease in humans, exhibited impaired intestinal motility and intestinal bacterial overgrowth by pro-inflammatory taxa including *Vibrio* spp. (126). This leads to increased intestinal inflammation characterized by elevated intestinal neutrophil infiltration and proinflammatory gene expression. The zebrafish model affords exciting opportunities to study intestinal motility and spatial patterns of microbiota colonization using high resolution *in vivo* imaging coupled with genetic manipulation of ENS function (127, 128).

## Future Directions and Perspectives

Comparison between innate immune processes in gnotobiotic zebrafish and mice have revealed extensive conservation between these host species (Table 3). This suggests that these host responses to the microbiota are ancient and were likely present in the last common ancestor over 420 million years ago (129). The emergence and evolutionary maintenance of these host responses to microorganisms illustrate that these are deeply important aspects of vertebrate biology, with zebrafish affording a unique experimental platform to study the dynamic host-microbe interface. Moving forward, the zebrafish is a model well-poised to provide insights of host-microbe interactions at several body sites, not just the intestine.

Studies in zebrafish have advanced our understanding of the effects of commensal microbiota colonization on host innate immune function. However, caution should be taken when making comparisons between published studies due to differences in developmental timepoints, host genetic background, transgenes used to monitor different cellular populations, as well as environmental variables such as nutrition, time of day, rearing temperature and media composition, all of which may impact microbiota-dependent and -independent host phenotypes. Moreover, while the small size of the larval zebrafish makes them amenable to *in vivo* imaging, this precludes longitudinal fecal and blood sampling, and restricts terminal tissue harvest to either whole animal or dissected digestive tracts (which can easily be contaminated by liver and pancreas in addition to the intestine due to the difficulty of precisely separating tissues manually). As a result many host responses have been evaluated from whole animal preparations, making it difficult to deconvolve the extent of variation in cell-type specific responses across distinct tissues and anatomical compartments. Moreover, as many innate immune lineages are lowly abundant (e.g., neutrophils typically represent ~1% of the total cell population in conventionally reared larvae at 6 dpf), bona fide leukocyte phenotypes may be lost due to signal-to-noise dilution in whole animal assessments. Fluorescence activated cell sorting (FACS) is one technological approach to overcome some of these limitations (31, 73). Additionally, the continued development of microdissection techniques using microneedles to sample small blood volumes or tissues will permit molecular analyse of distinct cellular populations from zebrafish larvae (130). These approaches, in combination with the advent of low-input molecular techniques (from PCR to single-cell sequencing), present exciting opportunities to parse the effects of the microbiota on rare and/or specific cell populations.

Molecular tools, such as antibodies, are underdeveloped in the zebrafish model, significantly limiting immunofluorescence and flow cytometry assays. Therefore, transgenesis has emerged as a powerful system for monitoring the spatiotemporal behavior and activation of specific cell populations *in vivo*. Numerous transgenic lines have been created to visualize and study specific immune cell types in zebrafish (Table 2). Due to the lack of existing reagents that specifically label some myeloid cell types, there are large knowledge gaps in understanding microbiota impacts on zebrafish innate immunity focusing on key immune cell types including mast cells and dendritic cells. Macrophage-specific promoter sequences (from genes including *fms, mpeg1, mfap4*, and *irg1*) have been used to drive a variety of transgenic reporters (57, 58, 131, 132). Though the impact of microbiota on zebrafish macrophage biology lags far behind that of neutrophils, there are reports of a reduction of *mpeg1*^+^ macrophages in the distal intestine of colonized 5 dpf larvae (35) (Table 2).

Despite the utility of transgenes in zebrafish, it is important to consider that the use of small promoter elements to engineer transgenic zebrafish may also led to pleiotropic labeling and varied lineage specificity of existing reagents. Moreover, these reagents are inherently transcriptional reporters which might exhibit dynamic behaviors following microbial colonization, injury, or infection (28, 133) (Table 2). In the future, the application of targeted knock-in methods to insert fluorescent proteins or tags directly into a genomic locus of interest may more faithfully recapitulate endogenous expression patterns of these genes.

Previous reports have focused on microbiota-dependent effects on innate immunity from zebrafish between 3 and 8 dpf. Current dogma supports a model whereby zebrafish larvae are initially exposed to environmental microorganisms at 3 dpf after hatching from their chorions. Thus, in designing experiments it may be prudent to include assays representative of later stages of larval development to allow for the establishment of microbiotas within the host (24). However, it is formally possible that MAMPs may be able to penetrate the chorion at earlier stages of zebrafish development (prior to hatching at 3 dpf). A number of recent studies in zebrafish have shown that inflammatory signaling pathways are important in promoting the niche for the development of HSCs (134, 135). This raises the possibility that early life exposures to microbial cues may shape host innate immunity, including the development of the hematopoietic compartment. While recent studies have begun to explore the effects of microbial exposure at other timepoints (as early as 1 dpf) (63), associated immunological phenotypes remain unexplored. Conversely, analysis of microbiota-dependent immune phenotypes during juvenile and adult stages of the zebrafish lifecycle remain almost completely unexplored. Methods to rear zebrafish GF past 2 weeks of life remain technically challenging but would provide valuable insights into microbial influence on zebrafish adaptive immune development as well as metamorphosis (27).

Variation in experimental results using the zebrafish model may also be attributed to underlying differences in microbiota composition across zebrafish facilities (21). Colonization of gnotobiotic zebrafish with defined consortia of bacteria could provide a standardized community that may offer a deeper understanding of taxa-specific impacts on vertebrate innate immune development and could improve experimental consistency and reproducibility.

Zebrafish offer unprecedented potential for experimental dissection of the bacterial products and signals that contribute to systemic innate immunity in the intact physiologic context of a living vertebrate. Using established techniques, it is now feasible to introduce bacterial mutant libraries or panels of bacterial strains into gnotobiotic zebrafish larvae to begin to disentangle the mechanisms by which the microbiota influence host innate immunity in medium-throughput screens. Zebrafish larvae mono- or poly-associated with defined bacterial taxa can be evaluated for effects on leukocyte responses during homeostasis or following challenge (e.g., recruitment to peripheral injury) by interrogating leukocyte abundance or activation (e.g., expression of inflammatory mRNAs). Further, coupling the genetic and pharmacological tractability of zebrafish with gnotobiotic manipulations allows for the identification and manipulation of genetic and molecular determinants of host-microbiota interactions *in vivo*. In combination, application of these medium-scale immersion strategies to gnotobiotic zebrafish will provide insights into host-microbiota-chemical interactions with relevance to toxins and pharmaceutical toxicity and metabolism (136). The emergence of organoid culture technology platforms derived from mammalian models presents exciting opportunities to monitor intestinal physiologies in a more scalable system, however these platforms do not provide the full anatomic and physiological complexity of the intact vertebrate digestive system afforded in the zebrafish. The optical transparency and numerous transgenic reporter lines available in zebrafish can be coupled with medium-scale imaging approaches to monitor host responses, unearthing novel interactions in the context of a whole living organism.

## Author Contributions

CM and JR: conceptualization. CM: writing—original draft. CM and JR: writing—review and editing, funding acquisition. JR: supervision.

### Conflict of Interest Statement

The authors declare that the research was conducted in the absence of any commercial or financial relationships that could be construed as a potential conflict of interest. The handling editor declared a past co-authorship with one of the authors JR.

## References

[1] Belkaid Y Hand TW. Role of the microbiota in immunity and inflammation. Cell. (2014) 157:121–41. 10.1016/j.cell.2014.03.011 24679531 PMC4056765

[2] McFall-Ngai M Hadfield MG Bosch TC Carey HV Domazet-Loso T Douglas AE. Animals in a bacterial world, a new imperative for the life sciences. Proc Natl Acad Sci USA. (2013) 110:3229–36. 10.1073/pnas.1218525110 23391737 PMC3587249

[3] Kawasaki T Kawai T. Toll-like receptor signaling pathways. Front Immunol. (2014) 5:461. 10.3389/fimmu.2014.00461 25309543 PMC4174766

[4] Wells JM Brummer RJ Derrien M MacDonald TT Troost F Cani PD. Homeostasis of the gut barrier and potential biomarkers. Am J Physiol Gastrointest Liver Physiol. (2017) 312:G171–93. 10.1152/ajpgi.00048.2015 27908847 PMC5440615

[5] Turvey SE Broide DH. Innate immunity. J Allergy Clin Immunol. (2010) 125 (2 Suppl. 2):S24–32. 10.1016/j.jaci.2009.07.016 19932920 PMC2832725

[6] Mitroulis I Ruppova K Wang B Chen LS Grzybek M Grinenko T. Modulation of myelopoiesis progenitors is an integral component of trained immunity. Cell. (2018) 172:147–161 e12. 10.1016/j.cell.2017.11.034 29328910 PMC5766828

[7] Netea MG Joosten LA Latz E Mills KH Natoli G Stunnenberg HG. Trained immunity: a program of innate immune memory in health and disease. Science. (2016) 352:aaf1098. 10.1126/science.aaf1098 27102489 PMC5087274

[8] Netea MG Schlitzer A Placek K Joosten LAB Schultze JL. Innate and adaptive immune memory: an evolutionary continuum in the host's response to pathogens. Cell Host Microbe. (2019) 25:13–26. 10.1016/j.chom.2018.12.006 30629914

[9] Gorjifard S Goldszmid RS. Microbiota-myeloid cell crosstalk beyond the gut. J Leukoc Biol. (2016) 100:865–79. 10.1189/jlb.3RI0516-222R 27605211 PMC6608064

[10] Thaiss CA Zmora N Levy M Elinav E. The microbiome and innate immunity. Nature. (2016) 535:65–74. 10.1038/nature18847 27383981

[11] Clarke TB. Microbial programming of systemic innate immunity and resistance to infection. PLoS Pathog. (2014) 10:e1004506. 10.1371/journal.ppat.1004506 25474680 PMC4256436

[12] Fiebiger U Bereswill S Heimesaat MM. Dissecting the interplay between intestinal microbiota and host immunity in health and disease: lessons learned from germfree and gnotobiotic animal models. Eur J Microbiol Immunol. (2016) 6:253–71. 10.1556/1886.2016.00036 27980855 PMC5146645

[13] Kennedy EA King KY Baldridge MT. Mouse microbiota models: comparing germ-free mice and antibiotics treatment as tools for modifying gut bacteria. Front Physiol. (2018) 9:1534. 10.3389/fphys.2018.01534 30429801 PMC6220354

[14] Kostic AD Howitt MR Garrett WS. Exploring host-microbiota interactions in animal models and humans. Genes Dev. (2013) 27:701–18. 10.1101/gad.212522.112 23592793 PMC3639412

[15] Parichy DM. Advancing biology through a deeper understanding of zebrafish ecology and evolution. Elife. (2015) 4:5635. 10.7554/eLife.05635 25807087 PMC4373672

[16] Engeszer RE Patterson LB Rao AA Parichy DM. Zebrafish in the wild: a review of natural history and new notes from the field. Zebrafish. (2007) 4:21–40. 10.1089/zeb.2006.9997 18041940

[17] Lickwar CR Camp JG Weiser M Cocchiaro JL Kingsley DM Furey TS. Genomic dissection of conserved transcriptional regulation in intestinal epithelial cells. PLoS Biol. (2017) 15:e2002054. 10.1371/journal.pbio.2002054 28850571 PMC5574553

[18] Rawls JF Samuel BS Gordon JI. Gnotobiotic zebrafish reveal evolutionarily conserved responses to the gut microbiota. Proc Natl Acad Sci USA. (2004) 101:4596–601. 10.1073/pnas.0400706101 15070763 PMC384792

[19] Bates JM Mittge E Kuhlman J Baden KN Cheesman SE Guillemin K. Distinct signals from the microbiota promote different aspects of zebrafish gut differentiation. Dev Biol. (2006) 297:374–86. 10.1016/j.ydbio.2006.05.006 16781702

[20] Brugman S Liu KY Lindenbergh-Kortleve D Samsom JN Furuta GT Renshaw SA. Oxazolone-induced enterocolitis in zebrafish depends on the composition of the intestinal microbiota. Gastroenterology. (2009) 137:1757–67 e1. 10.1053/j.gastro.2009.07.069 19698716

[21] Roeselers G Mittge EK Stephens WZ Parichy DM Cavanaugh CM Guillemin K. Evidence for a core gut microbiota in the zebrafish. ISME J. (2011) 5:1595–608. 10.1038/ismej.2011.38 21472014 PMC3176511

[22] Rawls JF Mahowald MA Ley RE Gordon JI. Reciprocal gut microbiota transplants from zebrafish and mice to germ-free recipients reveal host habitat selection. Cell. (2006) 127:423–33. 10.1016/j.cell.2006.08.043 17055441 PMC4839475

[23] Burns AR Stephens WZ Stagaman K Wong S Rawls JF Guillemin K. Contribution of neutral processes to the assembly of gut microbial communities in the zebrafish over host development. ISME J. (2016) 10:655–64. 10.1038/ismej.2015.142 26296066 PMC4817674

[24] Stephens WZ Burns AR Stagaman K Wong S Rawls JF Guillemin K. The composition of the zebrafish intestinal microbial community varies across development. ISME J. (2016) 10:644–54. 10.1038/ismej.2015.140 26339860 PMC4817687

[25] Wong S Stephens WZ Burns AR Stagaman K David LA Bohannan BJ. Ontogenetic differences in dietary fat influence microbiota assembly in the zebrafish gut. MBio. (2015) 6:e00687–15. 10.1128/mBio.00687-15 26419876 PMC4611033

[26] Pham LN Kanther M Semova I Rawls JF. Methods for generating and colonizing gnotobiotic zebrafish. Nat Protoc. (2008) 3:1862–75. 10.1038/nprot.2008.186 19008873 PMC2596932

[27] Melancon ES Gomez De La Torre Canny Sichel S Kelly M Wiles TJ Rawls JF. Best practices for germ-free derivation and gnotobiotic zebrafish husbandry. Methods Cell Biol. (2017) 138:61–100. 10.1016/bs.mcb.2016.11.005 28129860 PMC5568843

[28] Kanther M Tomkovich S Xiaolun S Grosser MR Koo J Flynn EJ 3rd. Commensal microbiota stimulate systemic neutrophil migration through induction of serum amyloid A. Cell Microbiol. (2014) 16:1053–67. 10.1111/cmi.12257 24373309 PMC4364439

[29] Rolig AS Parthasarathy R Burns AR Bohannan BJ Guillemin K. Individual members of the microbiota disproportionately modulate host innate immune responses. Cell Host Microbe. (2015) 18:613–20. 10.1016/j.chom.2015.10.009 26567512 PMC4701053

[30] Galindo-Villegas J Garcia-Moreno D de Oliveira S Meseguer J Mulero V. Regulation of immunity and disease resistance by commensal microbes and chromatin modifications during zebrafish development. Proc Natl Acad Sci USA. (2012) 109:E2605–14. 10.1073/pnas.1209920109 22949679 PMC3465450

[31] Murdoch CC Espenschied ST Matty MA Mueller O Tobin DM Rawls JF. Intestinal Serum amyloid A suppresses systemic neutrophil activation and bactericidal activity in response to microbiota colonization. PLoS Pathog. (2019) 15:e1007381. 10.1371/journal.ppat.1007381 30845179 PMC6405052

[32] Kanther M Sun X Muhlbauer M Mackey LC Flynn EJ 3rd Bagnat M. Microbial colonization induces dynamic temporal and spatial patterns of NF-kappaB activation in the zebrafish digestive tract. Gastroenterology. (2011) 141:197–207. 10.1053/j.gastro.2011.03.042 21439961 PMC3164861

[33] Bates JM Akerlund J Mittge E Guillemin K. Intestinal alkaline phosphatase detoxifies lipopolysaccharide and prevents inflammation in zebrafish in response to the gut microbiota. Cell Host Microbe. (2007) 2:371–82. 10.1016/j.chom.2007.10.010 18078689 PMC2730374

[34] Rolig AS Sweeney EG Kaye LE DeSantis MD Perkins A Banse AV. A bacterial immunomodulatory protein with lipocalin-like domains facilitates host-bacteria mutualism in larval zebrafish. Elife. (2018) 7:37172. 10.7554/eLife.37172 30398151 PMC6219842

[35] Koch BEV Yang S Lamers G Stougaard J Spaink HP. Intestinal microbiome adjusts the innate immune setpoint during colonization through negative regulation of MyD88. Nat Commun. (2018) 9:4099. 10.1038/s41467-018-06658-4 30291253 PMC6173721

[36] Rawls JF Mahowald MA Goodman AL Trent CM Gordon JI. *In vivo* imaging and genetic analysis link bacterial motility and symbiosis in the zebrafish gut. Proc Natl Acad Sci USA. (2007) 104:7622–7. 10.1073/pnas.0702386104 17456593 PMC1855277

[37] Qin C Zhang Z Wang Y Li S Ran C Hu J. EPSP of L. casei BL23 Protected against the infection caused by aeromonas veronii via enhancement of immune response in Zebrafish. Front Microbiol. (2017) 8:2406. 10.3389/fmicb.2017.02406 29375485 PMC5770644

[38] Zang L Ma Y Huang W Ling Y Sun L Wang X. Dietary Lactobacillus plantarum ST-III alleviates the toxic effects of triclosan on zebrafish (Danio rerio) via gut microbiota modulation. Fish Shellfish Immunol. (2019) 84:1157–69. 10.1016/j.fsi.2018.11.007 30423455

[39] Falcinelli S Picchietti S Rodiles A Cossignani L Merrifield DL Taddei AR. Lactobacillus rhamnosus lowers zebrafish lipid content by changing gut microbiota and host transcription of genes involved in lipid metabolism. Sci Rep. (2015) 5:9336. 10.1038/srep09336 25822072 PMC4378510

[40] Gioacchini G Giorgini E Olivotto I Maradonna F Merrifield DL Carnevali O. The influence of probiotics on zebrafish Danio rerio innate immunity and hepatic stress. Zebrafish. (2014) 11:98–106. 10.1089/zeb.2013.0932 24564619

[41] Zhang Z Ran C Ding QW Liu HL Xie MX Yang YL. Ability of prebiotic polysaccharides to activate a HIF1alpha-antimicrobial peptide axis determines liver injury risk in zebrafish. Commun Biol. (2019) 2:274. 10.1038/s42003-019-0526-z 31372513 PMC6658494

[42] Valenzuela MJ Caruffo M Herrera Y Medina DA Coronado M Feijoo CG. Evaluating the capacity of human gut microorganisms to colonize the zebrafish larvae (*Danio rerio*). Front Microbiol. (2018) 9:1032. 10.3389/fmicb.2018.01032 29896165 PMC5987363

[43] Girija V Malaikozhundan B Vaseeharan B Vijayakumar S Gobi NM. *In vitro* antagonistic activity and the protective effect of probiotic Bacillus licheniformis Dahb1 in zebrafish challenged with GFP tagged Vibrio parahaemolyticus Dahv2. Microb Pathog. (2018) 114:274–80. 10.1016/j.micpath.2017.11.058 29198821

[44] He S Ran C Qin C Li S Zhang H de Vos WM. Anti-infective effect of adhesive probiotic lactobacillus in fish is correlated with their spatial distribution in the intestinal tissue. Sci Rep. (2017) 7:13195. 10.1038/s41598-017-13466-1 29038557 PMC5643340

[45] Renshaw SA Trede NS. A model 450 million years in the making: zebrafish and vertebrate immunity. Dis Model Mech. (2012) 5:38–47. 10.1242/dmm.007138 22228790 PMC3255542

[46] Li Y Li Y Cao X Jin X Jin T. Pattern recognition receptors in zebrafish provide functional and evolutionary insight into innate immune signaling pathways. Cell Mol Immunol. (2017) 14:80–9. 10.1038/cmi.2016.50 27721456 PMC5214946

[47] Lam SH Chua HL Gong Z Lam TJ Sin YM. Development and maturation of the immune system in zebrafish, Danio rerio: a gene expression profiling, in situ hybridization and immunological study. Dev Comp Immunol. (2004) 28:9–28. 12962979 10.1016/s0145-305x(03)00103-4

[48] Kanther M Rawls JF. Host-microbe interactions in the developing zebrafish. Curr Opin Immunol. (2010) 22:10–9. 10.1016/j.coi.2010.01.006 20153622 PMC3030977

[49] Stachura DL Traver D Cellular dissection of zebrafish hematopoiesis Methods. Cell Biol. (2011) 101:75–110. 10.1016/B978-0-12-387036-0.00004-9 21550440

[50] Carradice D Lieschke GJ Zebrafish in hematology: sushi or science? Blood. (2008) 111:3331–42. 10.1182/blood-2007-10-052761 18182572 PMC2275003

[51] Gore AV Pillay LM Venero Galanternik M Weinstein BM. The zebrafish: a fintastic model for hematopoietic development and disease. Wiley Interdiscip Rev Dev Biol. (2018) 7:e312. 10.1002/wdev.312 29436122 PMC6785202

[52] Dobson JT Seibert J Teh EMS Da'as Fraser RB Paw BH. Carboxypeptidase A5 identifies a novel mast cell lineage in the zebrafish providing new insight into mast cell fate determination. Blood. (2008) 112:2969–72. 10.1182/blood-2008-03-145011 18635811

[53] Lugo-Villarino G Balla KM Stachura DL Banuelos K Werneck MB Traver D. Identification of dendritic antigen-presenting cells in the zebrafish. Proc Natl Acad Sci USA. (2010) 107:15850–5. 10.1073/pnas.1000494107 20733076 PMC2936643

[54] Balla KM Lugo-Villarino G Spitsbergen JM Stachura DL Hu Y Banuelos K. Eosinophils in the zebrafish: prospective isolation, characterization, and eosinophilia induction by helminth determinants. Blood. (2010) 116:3944–54. 10.1182/blood-2010-03-267419 20713961 PMC2981543

[55] Buchan KD Prajsnar TK Ogryzko NV de Jong NWM van Gent M Kolata J. A transgenic zebrafish line for *In vivo* visualisation of neutrophil myeloperoxidase. PLoS ONE. (2019) 14:e0215592. 10.1371/journal.pone.0215592 31002727 PMC6474608

[56] Hall C Flores MV Storm T Crosier K Crosier P. The zebrafish lysozyme C promoter drives myeloid-specific expression in transgenic fish. BMC Dev Biol. (2007) 7:42. 10.1186/1471-213X-7-42 17477879 PMC1877083

[57] Ellett F Pase L Hayman JW Andrianopoulos A Lieschke GJ. mpeg1 promoter transgenes direct macrophage-lineage expression in zebrafish. Blood. (2011) 117:e49–56. 10.1182/blood-2010-10-314120 21084707 PMC3056479

[58] Walton EM Cronan MR Beerman RW Tobin DM. The macrophage-specific promoter mfap4 allows live, long-term analysis of macrophage behavior during *Mycobacterial infection* in Zebrafish. PLoS ONE. 10:e0138949. 10.1371/journal.pone.0138949 26445458 PMC4596833

[59] Da'as S Teh EM Dobson JT Nasrallah GK McBride ER Wang H. Zebrafish mast cells possess an FcvarepsilonRI-like receptor and participate in innate and adaptive immune responses. Dev Comp Immunol. (2011) 35:125–34. 10.1016/j.dci.2010.09.001 20849876

[60] Da'as SI Coombs AJ Balci TB Grondin CA Ferrando AA Berman JN. The zebrafish reveals dependence of the mast cell lineage on Notch signaling *in vivo*. Blood. (2012) 119:3585–94. 10.1182/blood-2011-10-385989 22368273 PMC3375148

[61] Butko E Distel M Pouget C Weijts B Kobayashi I Ng K. Gata2b is a restricted early regulator of hemogenic endothelium in the zebrafish embryo. Development. (2015) 142:1050–61. 10.1242/dev.119180 25758220 PMC4360177

[62] Camp JG Frank CL Lickwar CR Guturu H Rube T Wenger AM. Microbiota modulate transcription in the intestinal epithelium without remodeling the accessible chromatin landscape. Genome Res. (2014) 24:1504–16. 10.1101/gr.165845.113 24963153 PMC4158762

[63] Phelps D Brinkman NE Keely SP Anneken EM Catron TR Betancourt D. Microbial colonization is required for normal neurobehavioral development in zebrafish. Sci Rep. (2017) 7:11244. 10.1038/s41598-017-10517-5 28894128 PMC5593827

[64] Kaiko GE Ryu SH Koues OI Collins PL Solnica-Krezel L Pearce EJ. The colonic crypt protects stem cell*s* from microbiota-derived metabolites. Cell. (2016) 165:1708–20. 10.1016/j.cell.2016.05.018 27264604 PMC5026192

[65] Cronan MR Tobin DM. Fit for consumption: zebrafish as a model for tuberculosis. Dis Model Mech. (2014) 7:777–84. 10.1242/dmm.016089 24973748 PMC4073268

[66] Meijer AH. Protection and pathology in TB: learning from the zebrafish model. Semin Immunopathol. (2016) 38:261–73. 10.1007/s00281-015-0522-4 26324465 PMC4779130

[67] Davison JM Lickwar CR Song L Breton G Crawford GE Rawls JF. Microbiota regulate intestinal epithelial gene expression by suppressing the transcription factor Hepatocyte nuclear factor 4 alpha. Genome Res. (2017) 27:1195–206. 10.1101/gr.220111.116 28385711 PMC5495071

[68] El Aidy S Merrifield CA Derrien M van Baarlen P Hooiveld G Levenez F. The gut microbiota elicits a profound metabolic reorientation in the mouse jejunal mucosa during conventionalisation. Gut. (2013) 62:1306–14. 10.1136/gutjnl-2011-301955 22722618

[69] Larsson E Tremaroli V Lee YS Koren O Nookaew I Fricker A. Analysis of gut microbial regulation of host gene expression along the length of the gut and regulation of gut microbial ecology through MyD88. Gut. (2012) 61:1124–31. 10.1136/gutjnl-2011-301104 22115825 PMC3388726

[70] Backhed F Ding H Wang T Hooper LV Koh GY Nagy A. The gut microbiota as an environmental factor that regulates fat storage. Proc Natl Acad Sci USA. (2004) 101:15718–23. 10.1073/pnas.0407076101 15505215 PMC524219

[71] Martinez-Guryn K Hubert N Frazier K Urlass S Musch MW Ojeda P. Small intestine microbiota regulate host digestive and absorptive adaptive responses to dietary lipids. Cell Host Microbe. (2018) 23:458–69 e5. 10.1016/j.chom.2018.03.011 29649441 PMC5912695

[72] Semova I Carten JD Stombaugh J Mackey LC Knight R Farber SA. Microbiota regulate intestinal absorption and metabolism of fatty acids in the zebrafish. Cell Host Microbe. (2012) 12:277–88. 10.1016/j.chom.2012.08.003 22980325 PMC3517662

[73] Espenschied ST Cronan MR Matty MA Mueller O Redinbo MR Tobin DM. Epithelial delamination is protective during pharmaceutical-induced enteropathy. Proc Natl Acad Sci USA. (2019) 116:16961–70. 10.1073/pnas.1902596116 31391308 PMC6708343

[74] Kiesler P Fuss IJ Strober W. Experimental models of inflammatory bowel diseases. Cell Mol Gastroenterol Hepatol. (2015) 1:154–70. 10.1016/j.jcmgh.2015.01.006 26000334 PMC4435576

[75] Marjoram L Alvers A Deerhake ME Bagwell J Mankiewicz J Cocchiaro JL. Epigenetic control of intestinal barrier function and inflammation in zebrafish. Proc Natl Acad Sci USA. (2015) 112:2770–5. 10.1073/pnas.1424089112 25730872 PMC4352795

[76] Oehlers SH Flores MV Hall CJ Crosier KE Crosier PS. Retinoic acid suppresses intestinal mucus production and exacerbates experimental enterocolitis. Dis Model Mech. (2012) 5:457–67. 10.1242/dmm.009365 22563081 PMC3380709

[77] Oehlers SH Flores MV Okuda KS Hall CJ Crosier KE Crosier PS. A chemical enterocolitis model in zebrafish larvae that is dependent on microbiota and responsive to pharmacological agents. Dev Dyn. (2011) 240:288–98. 10.1002/dvdy.22519 21181946

[78] Gkouskou KK Deligianni C Tsatsanis C Eliopoulos AG. The gut microbiota in mouse models of inflammatory bowel disease. Front Cell Infect Microbiol. (2014) 4:28. 10.3389/fcimb.2014.00028 24616886 PMC3937555

[79] Niess JH Adler G. Enteric flora expands gut lamina propria CX3CR1+ dendritic cells supporting inflammatory immune responses under normal and inflammatory conditions. J Immunol. (2010) 184:2026–37. 10.4049/jimmunol.0901936 20089703

[80] Reikvam DH Erofeev A Sandvik A Grcic V Jahnsen FL Gaustad P. Depletion of murine intestinal microbiota: effects on gut mucosa and epithelial gene expression. PLoS ONE. (2011) 6:e17996. 10.1371/journal.pone.0017996 21445311 PMC3061881

[81] Krishnan S Ding Y Saedi N Choi M Sridharan GV Sherr DH. Gut microbiota-derived tryptophan metabolites modulate inflammatory response in hepatocytes and macrophages. Cell Rep. (2018) 23:1099–111. 10.1016/j.celrep.2018.03.109 29694888 PMC6392449

[82] Kobayashi T Matsuoka K Sheikh SZ Russo SM Mishima Y Collins C. IL-10 regulates Il12b expression via histone deacetylation: implications for intestinal macrophage homeostasis. J Immunol. (2012) 189:1792–9. 10.4049/jimmunol.1200042 22786766 PMC3411910

[83] Ganal SC Sanos SL Kallfass C Oberle K Johner C Kirschning C. Priming of natural killer cells by nonmucosal mononuclear phagocytes requires instructive signals from commensal microbiota. Immunity. (2012) 37:171–86. 10.1016/j.immuni.2012.05.020 22749822

[84] Brown RL Sequeira RP Clarke TB. The microbiota protects against respiratory infection via GM-CSF signaling. Nat Commun. (2017) 8:1512. 10.1038/s41467-017-01803-x 29142211 PMC5688119

[85] Clarke TB. Early innate immunity to bacterial infection in the lung is regulated systemically by the commensal microbiota via nod-like receptor ligands. Infect Immun. (2014) 82:4596–606. 10.1128/IAI.02212-14 25135683 PMC4249320

[86] Clarke TB Davis KM Lysenko ES Zhou AY Yu Y Weiser JN. Recognition of peptidoglycan from the microbiota by Nod1 enhances systemic innate immunity. Nat Med. (2010) 16:228–31. 10.1038/nm.2087 20081863 PMC4497535

[87] Schuijt TJ Lankelma JM Scicluna BP de Sousa e Melo F Roelofs JJ de Boer JD. The gut microbiota plays a protective role in the host defence against pneumococcal pneumonia. Gut. (2016) 65:575–83. 10.1136/gutjnl-2015-309728 26511795 PMC4819612

[88] Hergott CB Roche AM Tamashiro E Clarke TB Bailey AG Laughlin A. Peptidoglycan from the gut microbiota governs the lifespan of circulating phagocytes at homeostasis. Blood. (2016) 127:2460–71. 10.1182/blood-2015-10-675173 26989200 PMC4874226

[89] Zhang D Chen G Manwani D Mortha A Xu C Faith JJ. Neutrophil ageing is regulated by the microbiome. Nature. (2015) 525:528–32. 10.1038/nature15367 26374999 PMC4712631

[90] Khosravi A Yanez A Price JG Chow A Merad M Goodridge HS. Gut microbiota promote hematopoiesis to control bacterial infection. Cell Host Microbe. (2014) 15:374–81. 10.1016/j.chom.2014.02.006 24629343 PMC4144825

[91] Balmer ML Schurch CM Saito Y Geuking MB Li H Cuenca M. Microbiota-derived compounds drive steady-state granulopoiesis via MyD88/TICAM signaling. J Immunol. (2014) 193:5273–83. 10.4049/jimmunol.1400762 25305320

[92] Deshmukh HS Liu Y Menkiti OR Mei J Dai N O'Leary CE. The microbiota regulates neutrophil homeostasis and host resistance to Escherichia coli K1 sepsis in neonatal mice. Nat Med. (2014) 20:524–30. 10.1038/nm.3542 24747744 PMC4016187

[93] Josefsdottir KS Baldridge MT Kadmon CS King KY. Antibiotics impair murine hematopoiesis by depleting the intestinal microbiota. Blood. (2017) 129:729–39. 10.1182/blood-2016-03-708594 27879260 PMC5301822

[94] Karmarkar D Rock KL. Microbiota signalling through MyD88 is necessary for a systemic neutrophilic inflammatory response. Immunology. (2013) 140:483–92. 10.1111/imm.12159 23909393 PMC3839652

[95] Wells JM Rossi O Meijerink M van Baarlen P. Epithelial crosstalk at the microbiota-mucosal interface. Proc Natl Acad Sci USA. (2011) 108(Suppl. 1):4607–14. 10.1073/pnas.1000092107 20826446 PMC3063605

[96] Hooper LV Wong MH Thelin A Hansson L Falk PG Gordon JI. Molecular analysis of commensal host-microbial relationships in the intestine. Science. (2001) 291:881–4. 10.1126/science.291.5505.881 11157169

[97] Liu T Zhang L Joo D Sun SC NF-kappaB signaling in inflammation. Signal Transduct Target Ther. (2017) 2. 10.1038/sigtrans.2017.23 PMC5661633 29158945

[98] van der Vaart M van Soest JJ Spaink HP Meijer AH Functional analysis of a zebrafish myd88 mutant identifies key transcriptional components of the innate immune system. Dis Model Mech. (2013) 6:841–54. 10.1242/dmm.010843 23471913 PMC3634667

[99] Burns AR Miller E Agarwal M Rolig AS Milligan-Myhre K Seredick S. Interhost dispersal alters microbiome assembly and can overwhelm host innate immunity in an experimental zebrafish model. Proc Natl Acad Sci USA. (2017) 114:11181–6. 10.1073/pnas.1702511114 28973938 PMC5651736

[100] Calo E Wysocka J. Modification of enhancer chromatin: what, how, and why? Mol Cell. (2013) 49:825–37. 10.1016/j.molcel.2013.01.038 23473601 PMC3857148

[101] Sommer F Nookaew I Sommer N Fogelstrand P Backhed F. Site-specific programming of the host epithelial transcriptome by the gut microbiota. Genome Biol. (2015) 16:62. 10.1186/s13059-015-0614-4 25887251 PMC4404278

[102] Darsigny M Babeu JP Dupuis AA Furth EE Seidman EG Levy E. Loss of hepatocyte-nuclear-factor-4alpha affects colonic ion transport and causes chronic inflammation resembling inflammatory bowel disease in mice. PLoS ONE. (2009) 4:e7609. 10.1371/journal.pone.0007609 19898610 PMC2764139

[103] Chellappa K Deol P Evans JR Vuong LM Chen G Briancon N. Opposing roles of nuclear receptor HNF4alpha isoforms in colitis and colitis-associated colon cancer. Elife. (2016) 5. 10.7554/eLife.10903 27166517 PMC4907689

[104] Consortium Uk Ibd Genetics Barrett JC Lee JC Lees CW Prescott NJ. Genome-wide association study of ulcerative colitis identifies three new susceptibility loci, including the HNF4A region. Nat Genet. (2009) 41:1330–4. 10.1038/ng.483 19915572 PMC2812019

[105] Jostins L Ripke S Weersma RK Duerr RH McGovern DP Hui KY. Host-microbe interactions have shaped the genetic architecture of inflammatory bowel disease. Nature. (2012) 491:119–24. 10.1038/nature11582 23128233 PMC3491803

[106] Marcil V Sinnett D Seidman E Boudreau F Gendron FP Beaulieu JF. Association between genetic variants in the HNF4A gene and childhood-onset Crohn's disease. Genes Immun. (2012) 13:556–65. 10.1038/gene.2012.37 22914433 PMC4931920

[107] Brand S Beigel F Olszak T Zitzmann K Eichhorst ST Otte JM. IL-22 is increased in active Crohn's disease and promotes proinflammatory gene expression and intestinal epithelial cell migration. Am J Physiol Gastrointest Liver Physiol. (2006) 290:G827–38. 10.1152/ajpgi.00513.2005 16537974

[108] Gewirtz AT Navas TA Lyons S Godowski PJ Madara JL. Cutting edge: bacterial flagellin activates basolaterally expressed TLR5 to induce epithelial proinflammatory gene expression. J Immunol. (2001) 167:1882–5. 10.4049/jimmunol.167.4.1882 11489966

[109] Roulis M Armaka M Manoloukos M Apostolaki M Kollias G Intestinal epithelial cells as producers but not targets of chronic TNF suffice to cause murine Crohn-like pathology. Proc Natl Acad Sci USA. (2011) 108:5396–401. 10.1073/pnas.1007811108 21402942 PMC3069201

[110] Schiering C Krausgruber T Chomka A Frohlich A Adelmann K Wohlfert EA. The alarmin IL-33 promotes regulatory T-cell function in the intestine. Nature. (2014) 513:564–8. 10.1038/nature13577 25043027 PMC4339042

[111] Shirota K LeDuy L Yuan SY Jothy S. Interleukin-6 and its receptor are expressed in human intestinal epithelial cells. Virchows Arch B Cell Pathol Incl Mol Pathol. (1990) 58:303–8. 1970694 10.1007/BF02890085

[112] von Moltke J Ji M Liang HE Locksley RM. Tuft-cell-derived IL-25 regulates an intestinal ILC2-epithelial response circuit. Nature. (2016) 529:221–5. 10.1038/nature16161 26675736 PMC4830391

[113] Dinarello CA. Immunological and inflammatory functions of the interleukin-1 family. Annu Rev Immunol. (2009) 27:519–50. 10.1146/annurev.immunol.021908.132612 19302047

[114] Dupaul-Chicoine J Yeretssian G Doiron K Bergstrom KS McIntire CR LeBlanc PM. Control of intestinal homeostasis, colitis, and colitis-associated colorectal cancer by the inflammatory caspases. Immunity. (2010) 32:367–78. 10.1016/j.immuni.2010.02.012 20226691

[115] Zaki MH Boyd KL Vogel P Kastan MB Lamkanfi M Kanneganti TD. The NLRP3 inflammasome protects against loss of epithelial integrity and mortality during experimental colitis. Immunity. (2010) 32:379–91. 10.1016/j.immuni.2010.03.003 20303296 PMC2982187

[116] Kolaczkowska E Kubes P. Neutrophil recruitment and function in health and inflammation. Nat Rev Immunol. (2013) 13:159–75. 10.1038/nri3399 23435331

[117] Kruger P Saffarzadeh M Weber AN Rieber N Radsak M von Bernuth H. Neutrophils: Between host defence, immune modulation, and tissue injury. PLoS Pathog. (2015) 11:e1004651. 10.1371/journal.ppat.1004651 25764063 PMC4357453

[118] Wang J. Neutrophils in tissue injury and repair. Cell Tissue Res. (2018) 371:531–9. 10.1007/s00441-017-2785-7 29383445 PMC5820392

[119] Renshaw SA Loynes CA Trushell DM Elworthy S Ingham PW Whyte MK. A transgenic zebrafish model of neutrophilic inflammation. Blood. (2006) 108:3976–8. 10.1182/blood-2006-05-024075 16926288

[120] Pase L Layton JE Wittmann C Ellett F Nowell CJ Reyes-Aldasoro CC. Neutrophil-delivered myeloperoxidase dampens the hydrogen peroxide burst after tissue wounding in zebrafish. Curr Biol. (2012) 22:1818–24. 10.1016/j.cub.2012.07.060 22940471

[121] Huang C Niethammer P. Tissue damage signaling is a prerequisite for protective neutrophil recruitment to microbial infection in Zebrafish. Immunity. (2018) 48:1006–13 e6. 10.1016/j.immuni.2018.04.020 29768163 PMC6082643

[122] de Oliveira S Lopez-Munoz A Candel S Pelegrin P Calado A Mulero V. ATP modulates acute inflammation *In vivo* through dual oxidase 1-derived H2O2 production and NF-kappaB activation. J Immunol. (2014) 192:5710–9. 10.4049/jimmunol.1302902 24842759

[123] Powell D Tauzin S Hind LE Deng Q Beebe DJ Huttenlocher A. Chemokine signaling and the regulation of bidirectional leukocyte migration in interstitial tissues. Cell Rep. (2017) 19:1572–85. 10.1016/j.celrep.2017.04.078 28538177 PMC5505660

[124] Lopez Nadal A Peggs D Wiegertjes GF Brugman S. Exposure to antibiotics affects saponin immersion-induced immune stimulation and shift in microbial composition in zebrafish larvae. Front Microbiol. (2018) 9:2588. 10.3389/fmicb.2018.02588 30420850 PMC6215861

[125] Goldberg RF Austen WG Jr Zhang X Munene G Mostafa G Biswas S. Intestinal alkaline phosphatase is a gut mucosal defense factor maintained by enteral nutrition. Proc Natl Acad Sci USA. (2008) 105:3551–6. 10.1073/pnas.0712140105 18292227 PMC2265168

[126] Rolig AS Mittge EK Ganz J Troll JV Melancon E Wiles TJ. The enteric nervous system promotes intestinal health by constraining microbiota composition. PLoS Biol. (2017) 15:e2000689. 10.1371/journal.pbio.2000689 28207737 PMC5331947

[127] Wiles TJ Jemielita M Baker RP Schlomann BH Logan SL Ganz J. Host gut motility promotes competitive exclusion within a model intestinal microbiota. PLoS Biol. (2016) 14:e1002517. 10.1371/journal.pbio.1002517 27458727 PMC4961409

[128] James DM Kozol RA Kajiwara Y Wahl AL Storrs EC Buxbaum JD. Intestinal dysmotility in a zebrafish (*Danio rerio*) shank3a;shank3b mutant model of autism. Mol Autism. (2019) 10:3. 10.1186/s13229-018-0250-4 30733854 PMC6357389

[129] Benton MJ Donoghue PC. Paleontological evidence to date the tree of life. Mol Biol Evol. (2007) 24:26–53. 10.1093/molbev/msl150 17047029

[130] Dickmeis T Feng Y Mione MC Ninov N Santoro M Spaink HP. Nano-sampling and reporter tools to study metabolic regulation in zebrafish. Front Cell Dev Biol. (2019) 7:15. 10.3389/fcell.2019.00015 30873407 PMC6401643

[131] Gray C Loynes CA Whyte MK Crossman DC Renshaw SA Chico TJ. Simultaneous intravital imaging of macrophage and neutrophil behaviour during inflammation using a novel transgenic zebrafish. Thromb Haemost. (2011) 105:811–9. 10.1160/TH10-08-0525 21225092

[132] Sanderson LE Chien AT Astin JW Crosier KE Crosier PS Hall CJ. An inducible transgene reports activation of macrophages in live zebrafish larvae. Dev Comp Immunol. (2015) 53:63–9. 10.1016/j.dci.2015.06.013 26123890

[133] Benard EL Racz PI Rougeot J Nezhinsky AE Verbeek FJ Spaink HP. Macrophage-expressed perforins mpeg1 and mpeg1.2 have an anti-bacterial function in zebrafish. J Innate Immun. (2015) 7:136–52. 10.1159/000366103 25247677 PMC6738794

[134] Espin-Palazon R Stachura DL Campbell CA Garcia-Moreno D Del Cid N Kim AD. Proinflammatory signaling regulates hematopoietic stem cell emergence. Cell. (2014) 159:1070–85. 10.1016/j.cell.2014.10.031 25416946 PMC4243083

[135] Li Y Esain V Teng L Xu J Kwan W Frost IM. Inflammatory signaling regulates embryonic hematopoietic stem and progenitor cell production. Genes Dev. (2014) 28:2597–612. 10.1101/gad.253302.114 25395663 PMC4248291

[136] National Academies of Sciences Engineering and Medicine Division on Earth and Life Studies Board on Life Sciences Board on Environmental Studies and Toxicology Committee on Advancing Understanding of the Implications of Environmental-Chemical Interactions with the Human Microbiome Environmental Chemicals, the Human Microbiome, and Health Risk: A Research Strategy. Washington, DC: National Academies Press (2017). 29431953

